# Electrochemical and computational estimations of cephalosporin drugs as eco-friendly and efficient corrosion inhibitors for aluminum in alkaline solution

**DOI:** 10.1038/s41598-022-17423-5

**Published:** 2022-08-03

**Authors:** Hanaa A. Mohamedien, Soha M. Kamal, Ahmed G. El-Deen, Mohamed Taha, Mohamed M. El-Deeb

**Affiliations:** 1 Applied Electrochemistry Laboratory, Chemistry Department, Faculty of Science, Beni-Suef University, Beni-Suef, 62511 Egypt; 2 Renewable Energy Science and Engineering Department, Faculty of Postgraduate Studies for Advanced Sciences (PSAS), Beni-Suef University, Beni-Suef, 62511 Egypt; 3 Materials Science and Nanotechnology Department, Faculty of Postgraduate Studies for Advanced Sciences (PSAS), Beni-Suef University, Beni-Suef, 62511 Egypt

**Keywords:** Corrosion, Molecular dynamics

## Abstract

In this study, the anionic state of Ceftriaxone sodium (Cefx) and Ceftazidime (Cefz) medication corrosion inhibition capabilities for Al in 0.1 M NaOH solution are explored using various electrochemical analyses. Furthermore, the morphological structure and surface chemical composition of the impact of these drugs on the Al substrate in NaOH are investigated. For the prediction and analysis of interactions between molecule structure and inhibition efficiency, quantum chemical calculations (QC), Monte Carlo simulations (MC), and molecular dynamics (MD) simulations (MD) are performed. The electrochemical findings reveal that the inhibitory effectiveness increases with increasing drug concentrations and declines with rising temperature, reaching a maximum value of 78.4% for 300 ppm Cefx while 59.5% for 300 ppm Cefz at 293 K, implying that Cefx outperforms for Cefz. In addition, the studied drugs act as cathodic inhibitors, and their adsorption is spontaneous and mixed type adsorption in its nature that obeys Freundlich isotherm for Cefz while Temkin isotherm is the best-fitted one for Cefx. Surface analysis and wettability measurements imply that Cefx and Cefz shield the Al against corrosion by surface adsorption and generating a protective hydrophobic film. Thermodynamic activation parameters in the absence and presence of 300 ppm of the studied drugs are calculated and discussed. The energies of the border molecular orbitals and computed molecular parameters for the investigated drugs revealed that anionic Cefx is more readily adsorbed on the Al surface than Cefz. This finding is validated further using MC and MD simulations. Overall, the proposed cephalosporin drugs delivered a cost-effective and facile approach for boosting the efficiency of corrosion inhibitors for Al under aggressive conditions.

## Introduction

Corrosion is a crucial issue for metals and their alloys because it produces significant economic losses and environmental problems. Aluminum is an essential metal in many industrial areas due to its several desirable properties, which include exceptional low density, thermal and electrical conductivity, high ductility, and abundance in nature^1^. It is utilized in vehicles, aircraft, household appliances, packaging, and electrical gadgets^2,3^. It resists corrosion due to its capacity to generate a natural oxide coating on its surface in various environments^4^. Because of the presence of hydroxide ions, which solubilize the amphoteric aluminum oxide protective layer, the alkaline media is the most corrosive for aluminum^5^.

Adding corrosion inhibitors to aluminum and its alloys in the aggressive media poses one of the efficient techniques to shield metals from corrosion, even though they slow down the rate of the corrosion process. Several studies have devoted on the developing of environmentally-friendly corrosion inhibitors with excellent inhibitory effectiveness^6–12^. Drugs have recently been employed as corrosion inhibitors for different metals owing to multiple benefits over traditional inhibitors that are toxic, hazardous to humans and other organisms, and have a negative impact on the environment^13–19^. Advantages of using drugs as corrosion inhibitors can be summarized into; (i) the presence of hetero-active centers in their molecular structures, (ii) their eco-friendly nature, and (iii) their highly molecular size^20^. There are two possible explanations for the corrosion protection provided by drug inhibitors: either an electrostatic attraction or chemical bonds formed with the metal's vacant orbitals^21^.

The second group of β-lactam antibiotics is known as cephalosporins, that are considered as the most effective broad-spectrum antibiotics and classified into five generations according to their antimicrobial properties^22^. Ceftriaxone sodium and ceftazidime drugs belong to the third generation of cephalosporins that were used for the corrosion protection technology^23–27^. Shukla and Quraishi studied the ceftriaxone drug as a green corrosion inhibitor for mild steel in HCl media^27^. They explained the ceftriaxone's inhibitory activity by its physical adsorption on steel substrate through β-lactam carbonyl and carboxylate group, and its adsorption following Langmuir isotherm. Singh et al. studied the inhibition efficiency of ceftazidime as an effective mild steel corrosion inhibitor in 1.0 M HCl media using electrochemical measurements, as well as AFM and SEM were used to investigate the microstructure^23^. The obtained data demonstrated that the tested drug with a concentration of 1.83 × 10^–4^ M decreased the metal roughness to 230 nm compared to 660 nm for the inhibitor-free solution.

Guo et al., introduced expired ceftriaxone sodium (Ceft), cefuroxime (Cefu) and cefotaxime sodium (Cefo) as corrosion inhibitors for carbon steel in 0.1 M H_2_SO_4_ solution^13^. Results showed that the order of the inhibition efficiency was Ceft > Cefo > Cefu due to the difference in their molecular structures.

Computational studies are performed to determine the relationship between the inhibitors' molecular structures and their inhibition efficiency^28,29^. The experimental findings of two antibacterial medicines, sulfamethoxazole (SZ) and norfloxacin(NF), were validated using quantum chemistry calculations and molecular dynamics simulations (MD)^30^. The inhibitory efficiency of NF was greater than that of SZ because the energy gap(ΔE) of NF was smaller than that of SZ. On the other hand, the calculated binding energy (E_binding_) of NF was relatively higher than that of SZ, reflecting the better stability of the created complex and increasing the inhibitory efficiency for NF compared to SZ.

**Figure 1:**
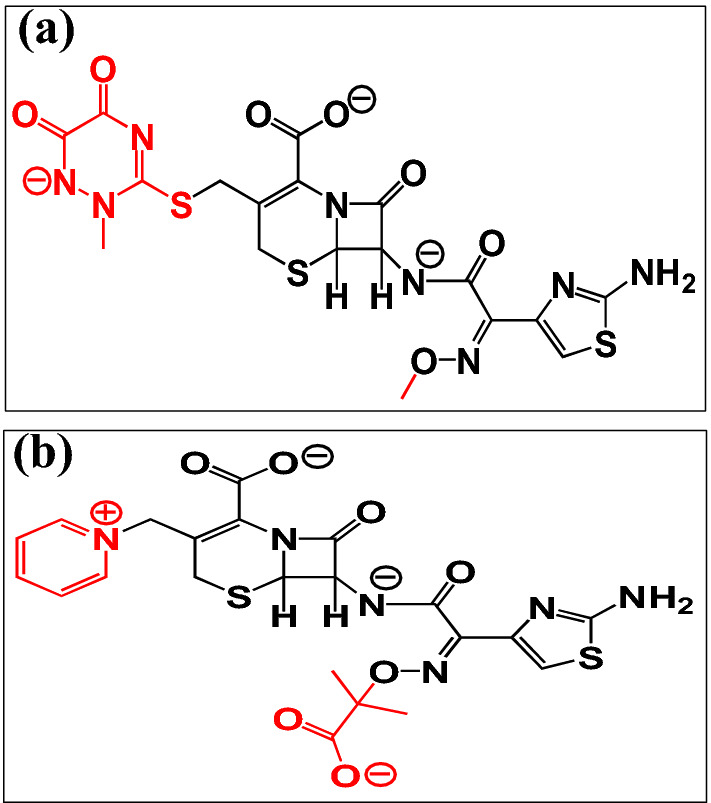
Structures of anionic (**a**) Cefx and (**b**) Cefz drugs in 0.1 M NaOH solution.

To the best of our knowledge, ceftriaxone sodium (Cefx), and ceftazidime (Cefz) drugs have never been applied as corrosion inhibitors for Al in NaOH media. Furthermore, there are few reports on the total suppression of aluminum corrosion in strong alkaline solutions owing to hydroxyl ion-specific adsorption on the metal surface. Therefore, our goal is to estimate the performance of these drugs to protect the vigorous corrosion of Al in 0.1 M NaOH media by electrochemical measurements, surface analysis and theoretical calculations. In addition, the inhibition protection will be correlated to their anionic structures as a result of the deprotonation of their functional groups in 0.1 M NaOH solutions^31–33^. The suggested drugs pave the way for developing a green and cost-effective corrosion inhibitors strategy for various industrial applications, including Al-air batteries, food and beverage packaging, and heavy sector implantation in the automotive, marine, and aerospace sectors. The structures of the studied anionic state of Cefx and Cefz are shown in Fig. 1.

## Experimental

### Materials and chemicals

The Cefx and Cefz are used as received from the Egyptian pharmaceutical store. Sodium hydroxide is provided from PIOCHEM chemical Co., Egypt. Al substrate as a working electrode was provided by (Naghammady Aluminum Co., Egypt) with the following chemical composition (wt%) 99.57% Al, 0.31% Fe, 0.07% Si, 0.015% Ti, 0.0016% Zn, 0.0003% Cr, 0.0019% Mn and 0.0007% Cu. Stock solutions of the studied drugs are prepared using doubled distilled H_2_O.

### Electrochemical

Electrochemical analyses are carried out using an electrochemical workstation (Origalys, OGF01A, France) in a glass cell with a standard three-electrode system, cleaned and polished Al metal (1 cm^2^) as working, Ag/AgCl and Pt wire as reference and counter electrodes, respectively. The obtained electrochemical results were recorded and analyzed using OrigaMaster-5 software. The potentiodynamic polarization curves are measured at a fixed potential of ± 100 mV around E_OCP_ at 293 K with a fixed sweep rate of 1 mV s^−1^. The electrochemical impedance spectroscopy (EIS) tests are performed at E_OCP_ using 10 mV amplitude, and the frequency range was 10 kHz to 100 mHz. The ZSimpWin software was utilized to fit the obtained results.

### Surface characterization

The morphological characteristics of Al substrates (1 cm × 1 cm × 0.1 cm) in the absence and existence of the examined drugs are investigated using FESEM (Zeiss Sigma 500 VP Analytical FE-SEM, Germany). In addition, the elemental composition of the surface is analyzed using XPS and elemental mappings coupled with the FESEM. XPS analyses are performed using (K-ALPHA, Thermo Fisher, USA). Al samples are polished, cleaned with acetone, rinsed with distilled water, and dried before being submerged in pure 0.1 M NaOH media and the presence of 300 ppm of each drug for 2 h at 293 K.

### Water contact angle measurements

The impact of Cefx and Cefz drugs on the surface wettability characteristics of Al surfaces are examined using the water contact angle (WCA) analysis. The Goniometer (model 250, Ramé-Hart, USA) was employed to estimate a sessile drop's static contact angle. Typically, 5–8 μL of aqueous solutions are dropped onto a polished and balanced Al surface using a microsyringe at room temperature. The resulted images are processed using DROP-image software to evaluate the contact angle (θ).

### Computational methodology

#### Hartree Fock calculations (HF)

Quantum chemical calculations (QC) depending on HF are carried out utilizing Gaussian 09 program^34^. The input files of the studied drugs are prepared with GaussView6.0.16. The structures of Cefx and Cefz are optimized by 6-311G++ (d, p) basis set. The energies of the boundary molecular orbitals (HOMO and LUMO) for the tested drugs are acquired and utilized to compute additional molecular properties such as ionization energy (I), global hardness (η), electron affinity (A), electronegativity (χ), and softness (σ) using the following equations^35,36^:

1 $${\text{I}} = - {\text{E}}_{{{\text{HOMO}}}}$$

2 $${\text{A }} = - {\text{ E}}_{{{\text{LUMO}}}}$$

3 $$\upchi = \left( {{\text{I }} + {\text{ A}}} \right)/{2}$$

4 $$\upeta = \left( {{\text{I }}-{\text{ A}}} \right)/{ 2}$$

5 $$\upsigma = 1/\upeta$$

The value of χ and η can be used to compute the proportion of electrons transported (ΔN) from the inhibitor molecule to the Al surface using Eq. (6)^35,36^.

6 $$\Delta {\text{N }} = \left( {\upchi_{{{\text{Al}}}} - \upchi_{{{\text{inh}}}} } \right)/2\left( {\upeta_{{{\text{Al}}}} + \upeta_{{{\text{inh}}}} } \right)$$

where χ_Al_ = 3.23 eV/mol and η_Al_ = 0 eV/mol, based on Pearson's electronegativity scale, supposing the I = A for the Al bulk structure owing to they are softer than neutral Al atoms^37^.

#### Monte Carlo simulation (MC)

MC simulation of the configurational space of a single molecule of the Cefx and Cefz with Al (111) surface is employed to determine low-energy adsorption sites as the temperature gradually reduces. The simulated annealing process used the Metropolis method. The MC simulation is carried out by the Adsorption Locator module that existed in the BIOVIA Materials Studio 2017 package. A simulation box (28.63 Å × 28.63 Å × 37.01 Å) with a 35 Å-thick vacuum slab above the surface is created from a supper cell of (5 × 5) using a unit cell of (4.05 Å × 4.05 Å × 4.05 Å) Al with a cell formula of Al4. The simulations are carried out using the COMPASS forcefield and its charges are used. The summation method for the electrostatic interaction is Ewald^38^. For the van der Waals interactions, a cut-off distance of 15.5 Å is used with a cubic spline switching function, and the summing approach is atom-based.

#### Molecular dynamics simulation (MD)

The MD simulations are conducted employing the FORCITE module implemented in the BIOVIA Materials Studio. Because corrosion occurs in aqueous solution, the lowest-energy structures of the inhibitor-Al complexes derived from the MC simulation are filled with water (950 molecules) to model the influence of solvent. The forcefield and its charges, as well as the summation method, are the same as in the MC simulations. The MD simulations are run at 298 K (controlled by the Nose thermostat) utilizing a canonical ensemble (*NVT*) with a time step of 1.0 fs, and a simulation time of 1000 ps (1 × 10^6^ steps).

## Results and discussions

### Open circuit potential (E_OCP_) measurements

**Figure 2:**
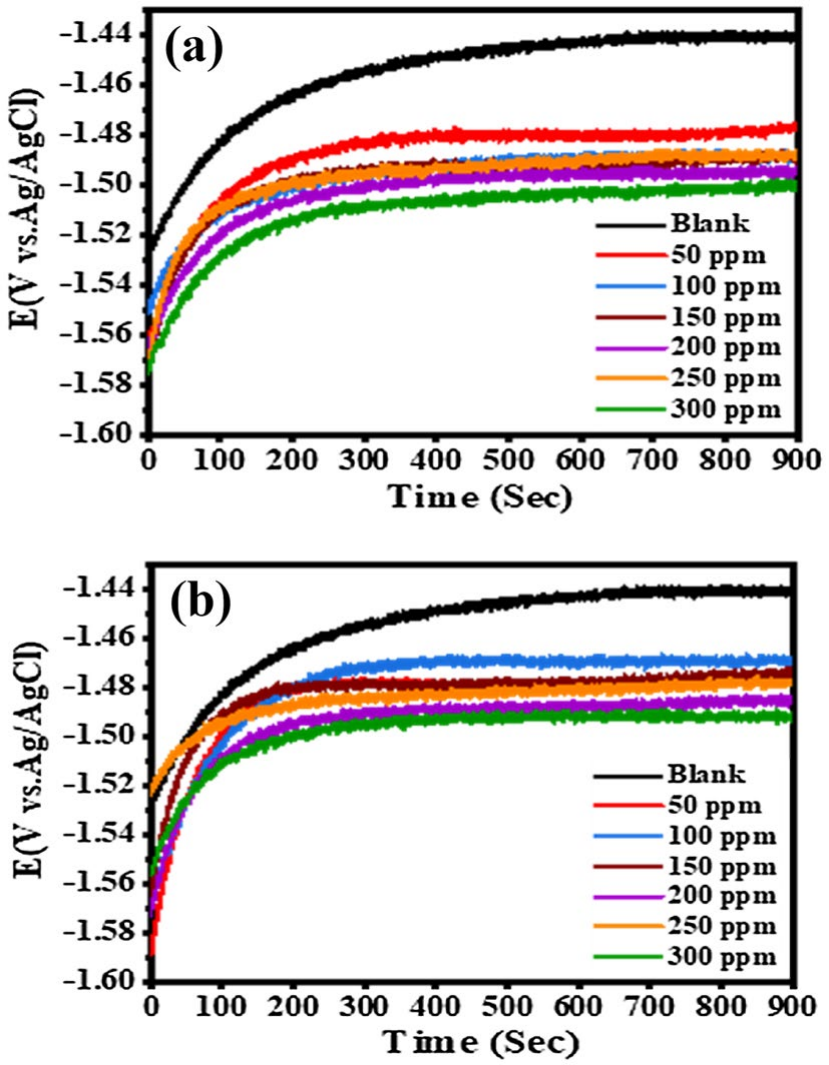
OCP curves of Al in 0.1 M NaOH solution containing different concentrations of (**a**) Cefx and (**b**) Cefz at 293 K.

The change in the E_OCP_ values of the Al electrode with immersion time in the pure 0.1 M NaOH media and existence of different concentrations of Cefx and Cefz is studied to emphasize the electrochemical properties in alkaline solutions at 293 K. Figure 2 depicts the E_OCP_ of the Al substrate as a function of the immersion duration in various concentrations of Cefx (Fig. 2a) and Cefz (Fig. 2b) ranged from 0 to 300 ppm in 0.1 M NaOH solution. The detailed E_OCP_ values, on the other hand, are listed in Table 1. As represented in the Fig. 2 that the E_OCP_ value in a blank solution shows a positive shift at the initial 400-s period, which can be explained by the anodic passivation caused by the formation of insoluble Al_2_O_3_ or Al(OH)_3_^39^. Then, when the immersion period is increased, it enters a stable state. The addition of Cefx and Cefz causes the E_OCP_ to move negatively compared to the blank solution, suggesting that the cathodic corrosion process is significantly slowed.

**Table 1:** Electrochemical kinetic parameters and the inhibition efficiencies of Al in 0.1 M NaOH solution in the absence and presence of different concentrations of Cefx and Cefz drugs at 293 K.

C (ppm)	−E_OCP_ (mV) (vs. Ag/AgCl)		−E_corr_ (mV) (vs. Ag/AgCl)		*i*_*corr*_ (μA/cm^2^)		βa (mV/d)		−βc (mV/d)		θ		*η*%	
	Cefx	Cefz	Cefx	Cefz	Cefx	Cefz	Cefx	Cefz	Cefx	Cefz	Cefx	Cefz	Cefx	Cefz
0	1455		1463.7		212.29		420.5		258		–		–	
50	1488	1485	1504.8	1497.9	168.81	164.54	253.1	199.7	345.5	305.4	0.205	0.225	20.5	22.5
100	1496	1479	1521.1	1497.2	104.1	157.46	168.5	281.1	177.3	281.3	0.51	0.258	51	25.8
150	1497	1482	1511	1480.1	100.75	155.43	126.5	212.4	119	155.4	0.525	0.268	52.5	26.8
200	1503	1494	1534.1	1497.7	72.92	130.19	122.4	200	103.5	140.9	0.656	0.387	65.6	38.7
250	1489	1485	1497.8	1498.1	70.94	107.73	74.7	180.3	67.5	187.7	0.666	0.493	66.6	49.3
300	1511	1498	1533.4	1512.2	45.78	85.99	72.9	125.5	84.2	111.2	0.784	0.595	78.4	59.5

The positive shift in the beginning of the curve for the inhibited solutions occurs till 200 s period indicating that the studied drugs cover the Al surface and hence form protective film on Al surface^40,41^. The inhibition of the cathodic reaction causes accumulation of the electrons generated from the anodic reaction, leading to more negative potential values for inhibited solutions compared to blank solution^40^.

**Figure 3:**
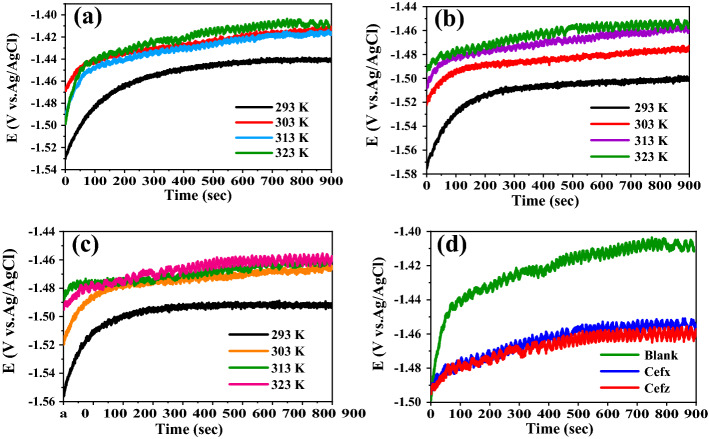
OCP curves of Al in 0.1 M NaOH solution in the absence and presence of 300 ppm of Cefx and Cefz drugs at different temperatures: (**a**) blank, (**b**) Cefx, (**c**) Cefz and (**d**) Blank, Cefx and Cefz at 323 K.

The E_OCP_ are also analyzed at 303, 313 and 323 K in the absence and existence of 300 ppm of the investigated drugs and their values are listed in Table 2. It is clearly shown from Fig. 3a–c that the positive shift in the beginning of the curves at high temperature is less than that at 293 K, indicating that the time taken for the formation of passive layer before reaching the steady-state value declines and therefore the inhibition efficiency decreases. In addition, the E_OCP_ curves at high temperatures are shifted to positive direction compared to that at 293 K either for blank or both studied drugs. Figure 3d represents the OCP curves of blank and 300 ppm of the studied drugs at 323 k. The figure indicates that the Curves in presence of both Cefx and Cefz drugs are remained in negative direction relative to blank one at the same temperature indicating that increasing the temperature doesn't alter the mechanism of inhibition or type of used inhibitor.

**Table 2:** Electrochemical and thermodynamic kinetic parameters and inhibition efficiencies of Al in 0.1 M NaOH solution based on PDP in the absence and presence of 300 ppm Cefx and Cefz drugs at different temperatures.

	T (K)	βa (mV/d)	−βc (mV/d)	−E_OCP_ (mV)	−E_corr_ (mV)	i_corr_ (µA/cm^2^)	θ	*η*%	Ea (kJ/mol)	ΔH* (kJ/mol)	ΔS* (kJ/mol K)
Blank	293	420.5	258	1455	1463.7	212.29	–	–	14.59	12.03	− 159.83
	303	221.2	253.8	1427	1452	228.83					
	313	123.8	152	1431	1447.6	238.6					
	323	200.3	188.1	1422	1435.1	392.1					
Cefx	293	72.9	84.2	1511	1533.4	45.78	0.784	78.4	41.82	39.26	− 78.49
	303	74.6	68	1485	1497.1	96.86	0.577	57.7			
	313	72	74.5	1470	1478.6	134.3	0.438	43.8			
	323	97.7	105.5	1464	1480.4	240.8	0.386	38.6			
Cefz	293	125.5	111.2	1498	1512.2	85.99	0.595	59.5	33.16	30.6	− 104.19
	303	93.5	94	1475	1490.5	106.04	0.537	53.7			
	313	103.2	99.1	1469	1473.9	172.25	0.278	27.8			
	323	121.7	132.2	1467	1478.1	300.5	0.234	23.4

### Potentiodynamic polarization (PDP) measurements and the mechanism

**Figure 4:**
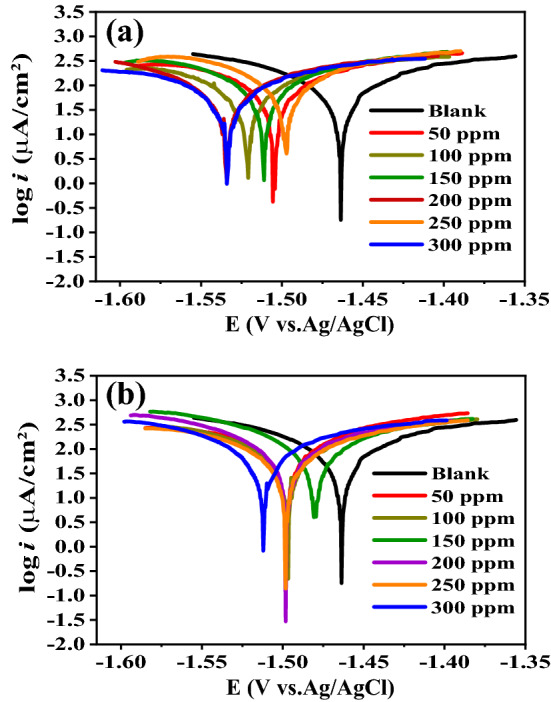
Potentiodynamic polarization curves of Al in 0.1 M NaOH solution containing different concentrations of (**a**) Cefx and (**b**) Cefz at 293 K.

PDP tests are performed on Al in 0.1 M NaOH medium by automatically altering the potential ± 100 mV against its E_OCP_ value in the absence and existence of various concentrations (50–300 ppm) of Cefx and Cefz at 293 K with a sweep rate of 1 mV/s. Table 1 lists the electrochemical polarization characteristics, including corrosion potentials (*E*_corr_), corrosion current densities (*i*_corr_), anodic Tafel slopes (*β*a) and cathodic Tafel slopes (*β*c). The inhibition efficiencies (*η*%) are obtained using the equation below^42,43^.

7 $$\eta \% = \left[ {\left( {{\text{i}}^{{\text{o}}}{_{{{\text{corr}}}}} - {\text{i}}_{{{\text{corr}}}} } \right)/{\text{i}}^{{\text{o}}}{_{{{\text{corr}}}}} } \right] \times 100$$

where i^o^_corr_ and i_corr_ are the corrosion current densities for blank and inhibited solutions, respectively. Figure 4 represents the PDP profiles of the Al in the pure 0.1 M NaOH and existence of different concentrations of Cefx (Fig. 4a) and Cefz (Fig. 4b). The results demonstrate that the greater value of I_corr_ and the more positive value of the E_corr_ (212.29 µA/cm^2^, − 1463.7 mV vs. Ag/AgCl) are observed in the inhibitor-free solution compared to (45.78 µA/cm^2^, − 1533.4 mV vs. Ag/AgCl) and (85.99 µA/cm^2^, − 1512.2 mV vs. Ag/AgCl) in the presence of 300 ppm from Cefx and Cefz, respectively. Furthermore, the values of E_corr_ for all studied concentrations of both drugs are slightly shifted to negative direction compared to inhibitor-free solution, this means that the used drugs are cathodic ones, that mainly inhibit the cathodic reduction reaction of H_2_O through the retardation of the cathodic polarization and decreasing the corrosion current density without any effect on the anodic polarization^44^. The multistep electrochemical process of anodic dissolution of Al in the alkaline solution has been summarized as follows^40,45^:

8 $${\text{Al}}\left( {{\text{ss}}} \right) + {\text{ OH}}^{ - } \to {\text{Al}}\left( {{\text{OH}}} \right)_{{{\text{ads}}}} + {\text{ e}}^{ - }$$

9 $${\text{Al}}\left( {{\text{OH}}} \right)_{{{\text{ads}}}} + {\text{ OH}}^{ - } \to {\text{Al}}\left( {{\text{OH}}} \right)_{{{2},{\text{ads}}}} + {\text{ e}}^{ - }$$

10 $${\text{Al}}\left( {{\text{OH}}} \right)_{{{2},{\text{ads}}}} + {\text{ OH}}^{ - } \to {\text{Al}}\left( {{\text{OH}}} \right)_{{{3},{\text{ads}}}} + {\text{ e}}^{ - }$$

The final step is the conversion of Al(OH)_3,ads_ to soluble aluminate species leaving a bare surface site (ss)

11 $${\text{Al}}\left( {{\text{OH}}} \right)_{{{3},{\text{ads}}}} + {\text{ OH}}^{ - } \to {\text{Al}}\left( {{\text{OH}}} \right)_{{4}}^{ - } + {\text{ ss}}$$

On the surface of aluminum, the cathodic process includes the reduction of water molecules^45,46^.

12 $${\text{ss }} + {\text{ H}}_{{2}} {\text{O }} + {\text{ e}}^{ - } \to {\text{H}}_{{{\text{ads}}}} + {\text{ OH}}^{ - }$$

13 $${\text{H}}_{{{\text{ads}}}} + {\text{ H}}_{{2}} {\text{O }} + {\text{ e}}^{ - } \to {\text{H}}_{{{2}({\text{gas}})}} + {\text{ OH}}^{ - } + {\text{ ss}}$$

**Figure 5:**
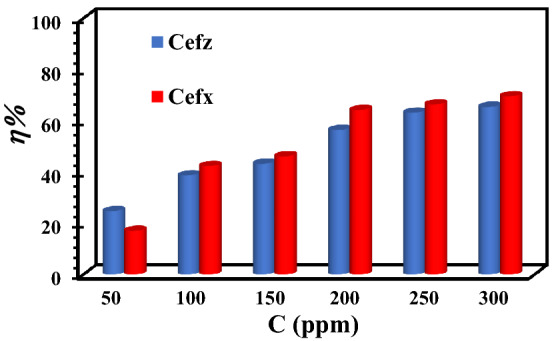
Variation of the inhibition efficiencies vs. inhibitor concentrations depending on the potentiodynamic polarization measurements at 293 K.

Figure 5 exhibits the variation of the calculated values of *η*% of Cefx and Cefz as a function of their concentrations. The findings reveal that *η*% values enhance with increasing the concentrations of the tested drugs. The maximum inhibition efficiency (78.4%) is detected at 300 ppm of Cefx, concerning 59.5% at 300 ppm of Cefz. The analyses show that increasing the concentrations of Cefx and Cefz improves the Al surface coverage and hence the inhibition efficiency in the sequence Cefx > Cefz. Due to their adsorption via their hetero-active sites on the Al surface, these drugs produce a barrier protective film that inhibits the active sites for reduction reactions^47^.

**Figure 6:**
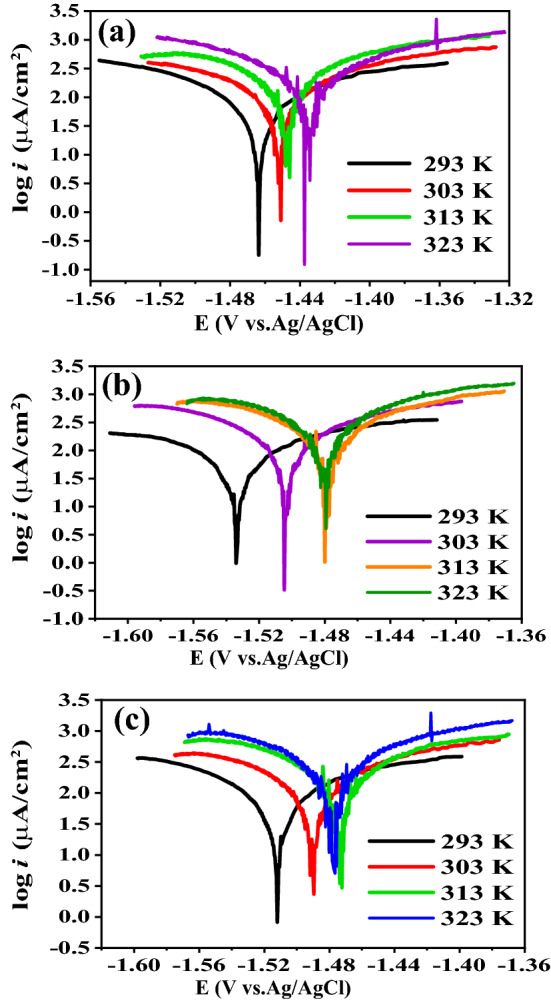
PDP curves of Al in 0.1 M NaOH solution in the absence and presence of 300 ppm of Cefx and Cefz drugs at different temperatures: (**a**) blank, (**b**) Cefx and (**c**) Cefz.

PDP measurements are also accomplished at 303,313 and 323 K in the absence and presence 300 ppm of Cefx and Cefz drugs. The electrochemical polarization parameters such as *i*_corr_, *E*_corr_, *β*c and *β*a are given in Table 2. Figure 6 represents PDP of the Al in pure 0.1 M NaOH and presence of 300 ppm of Cefx and Cefz drugs at various temperatures from 293 to 323 K. As demonstrated in the Fig. 6, the E_corr_ at high temperatures is shifted to positive direction relative to that at 293 K which implies that the studied drugs act as cathodic inhibitors even at high temperatures. Increasing the temperatures results in the desorption of Cefx and Cefz anions from Al surface so the inhibition of cathodic reaction decreases, accumulation of electrons at the anode reduces and thus the potential moves to positive direction. Table 2 strongly suggests that raising the temperature increases the corrosion current (i_corr_), lowering the inhibitory efficiency of both Cefx and Cefz drugs.

### Electrochemical impedance spectroscopy (EIS) measurements

**Figure 7:**
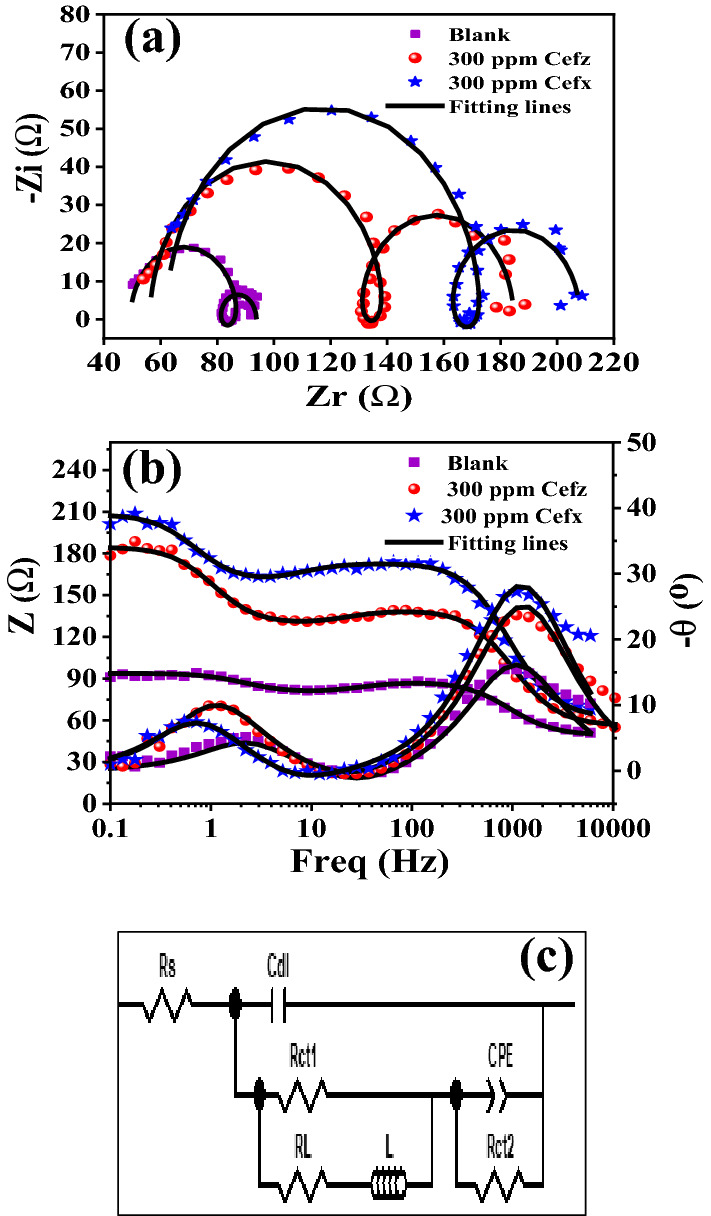
Electrochemical impedance spectroscopy measurements of Al in 0.1 M NaOH solution at E_OCP_ in the absence and presence of 300 ppm of Cefx and Cefz at 293 K, (**a**) Nyquist plots, (**b**) Bode plots and (**c**) electrochemical equivalent circuit (EC_1_).

Aluminum/electrolyte interface relationship during the corrosion process is evaluated using EIS technique at different concentrations of both Cefx and Cefz at E_OCP_. Figure 7a depicts the Nyquist plots of Al in blank 0.1 M NaOH media and existence of 300 ppm of both Cefx and Cefz at 293 K. The impedance profile displays the presence of two capacitive loops at high and low-frequency zones, which are ascribed to charge transfer reactions through the Al/electrolyte interface, as well as the presence of one inductive loop at the intermediate frequency region for the adsorption of the corrosion product intermediates on Al surface^46,48^. However, in the presence of both drugs, the capacitive loops' width grows without altering the impedance diagrams' forms, indicating that the corrosion process is unchanged in the absence and presence of the examined drugs^49^.This observation agrees well with PDP measurements which can be indicated from the parallel cathodic polarization curves^44^. At high frequencies, the large capacitive semicircle is referred to as the redox Al to Al^+^ reaction (Eq. 8) which is the rate-determining phase in the charge transfer reaction throughout the corrosion process^48,49^. At low frequencies, the second capacitive semicircle can be attributed to the fast-complementary redox Al^+^ to Al^3+^ reactions (Eqs. 9 and 10). Meanwhile, the inductive loop in the middle frequencies is assigned to the presence of the adsorbed Al(OH)_1–3_ intermediates on Al surface during its dissolution^48,49^. The corresponding phase angle and Bode impedance magnitude plots for Al in pristine 0.1 M NaOH solution and existence of 300 ppm of the Cefx and Cefz at 293 K are depicted in Fig. 7b. The bode graphs indicate the presence of two-time constants for charge transfer events during Al dissolution. Furthermore, the area under the curves is ordered as follows: Cefx > Cefz > blank. This discovery may be illustrated by the adsorption of the examined medicines on the Al surface, which protects it against corrosion. Furthermore, the higher area in the presence of Cefx compared to Cefz suggesting the Al surface coverage in case of Cefx is higher than that of Cefz, consequently its higher inhibition efficiency.

Figure 7c depicts a model of the experimental data fitted to an electrochemical equivalent circuit, which is consistent with recent work by Yong Liu et al.^49^.

According to equivalent circuit measurements, the electrochemical parameters including, resistance to charge transfer (R_ct1_, R_ct2_), bulk resistance (R_s_), and double-layer (C_dl_) in pure NaOH electrolyte and in existence of varying concentrations of Cefx and Cefz are summarized in Table 3. Data show that the presence of Cefx and Cefz changes the structure of the Al electrolyte interface compared to inhibitor free solution. This behaviour is resulted from increasing R_s_, R_ct1_ and R_ct2_ values with decreasing C_dl_ and CPE values in the presence of the studied drugs compared to the inhibitor free solution. These findings can be explained to the replacement of water molecules by the adsorbed barrier protective insulating layers from Cefx and Cefz on Al surface, which decreasing the local dielectric constant and/or increasing the thickness of the adsorbed barrier protective layer, that impedes the charge transfer reaction through Al/electrolyte interface^47^. The inhibition efficiencies (*η*%) are calculated from total charge transfer resistance R_T_ (R_T_ = R_ct1_ + R_ct2_) and listed in Table 3 using the following equation^48^:

14 $$\eta \% = \uptheta \times 100$$

15 $$\uptheta = \left( {{\text{R}}_{{\text{T}}} - {\text{ R}}_{{\text{T}}}{^{{\text{o}}}} } \right)/{\text{ R}}_{{\text{T}}}$$

where θ is the surface coverage, R_T_^o^ and R_T_ are total charge transfer resistances in the absence and presence of the studied drugs, respectively. Data show that, the values of *η*% increase with increasing the concentrations of both drugs in the following order: Cefz < Cefx and reach the maximum (69.6%) at 300 ppm for Cefx which agreed well with PDP measurements.

**Table 3:** Electrochemical parameters and the inhibition efficiencies of Al in 0.1 M NaOH solution based on EIS measurements in the absence and presence of different concentrations of Cefx and Cefz drugs at 293 K.

	C (ppm)	Rs (Ω)	C (μF)	R_ct1_ (Ω)	Q-Y_0_ (mF)	n	R_ct2_ (Ω)	θ	*η*%
Blank	0	49.35	5.131	30.65	5.437	1	13.8	–	–
Cefx	50	49.72	5.558	37.84	5.955	1	15.73	0.1702	17.02
	100	55.43	3.074	53.11	5.215	1	23.91	0.4228	42.28
	150	58.95	2.397	58.09	5.298	1	24.36	0.4608	46.08
	200	63.72	1.971	79.17	4.12	1	45.03	0.642	64.2
	250	54.87	1.706	79.02	3.081	1	53.26	0.664	66.4
	300	61.98	1.959	97.12	4.850	1	49.03	0.696	69.6
Cefz	50	52.5	2.94	37.11	3.286	0.6797	21.93	0.2471	24.71
	100	49.52	2.496	46.31	4.46	1	26.08	0.3859	38.59
	150	51.6	2.623	47.77	3.910	1	30.52	0.4322	43.22
	200	62.98	2.45	61.55	4.624	0.953	40.4	0.564	56.4
	250	64.34	2.261	68.49	3.416	0.8	51.97	0.631	63.1
	300	56.23	2.357	73.17	2.955	1	55.25	0.654	65.4

**Figure 8:**
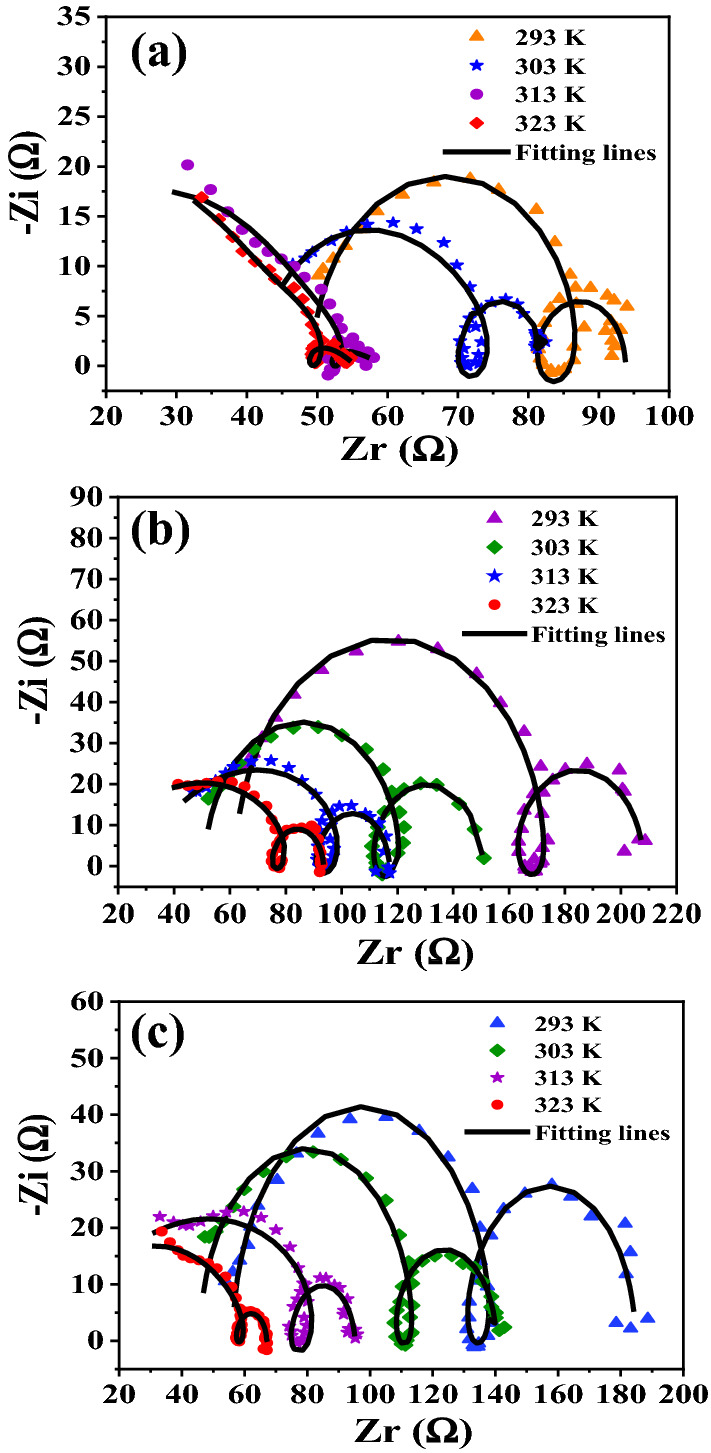
Nyquist plots for Al immersed in 0.1 M NaOH in the absence and presence of 300 ppm of Cefx and Cefz drugs at different temperatures (from 293 to 323 K): (**a**) Blank, (**b**) Cefx and (**c**) Cefz.

EIS measurements are also performed at 303,313 and 323 K in the absence and presence 300 ppm of Cefx and Cefz drugs at E_OCP_. Data obtained at 303 K are fitted to the same equivalent circuit which fits 293 K data (assume EC_1_) while this EC_1_ doesn't fit the data obtained at 313 and 323 K (assume EC_2_), therefore C_dl_ in EC_1_ is replaced by CPE in EC_2_. The electrochemical parameters derived from EC_2_ (Fig. S1), including constant phase element (CPE1 and CPE2), charge transfer resistances (*R*_ct1_, R_ct2_), solution resistance (R_s_), calculated R_T_ and η% are given in Table 4. It is clearly shown from Fig. 8 that the shape of high frequency capacitive loop changes which is proved by the replacement of the C_dl_ in EC_1_ (Fig. 7c) for Al samples at 293 and 303 K by CPE in EC_2_ (Fig. S1a) at 313 and 323 K which is elucidated to the desorption of Cefx and Cefz anions from Al surface at high temperatures and thus increase the corrosion rate and consequently the inhomogeneity of Al surface. It is clear from Table 4 that the addition of the studied drugs to sodium hydroxide solution at any given temperature rises the value of R_T_ but reduces the value of C_dl_ compared to blank solution at the same temperature. The decrease in R_T_ values of Cefx and Cefz drugs with increasing the temperature confirms the desorption of the studied drugs from Al surface, decreasing the Al surface coverage with these drugs, increasing the corrosion rate and therefore decrease the inhibition efficiencies. Bode plots are shown in Fig. S1b–d. It is clearly seen that the impedance (Z) of Al in the absence and presence of Cefx and Cefz drugs at 293 and 303 K is more at lower frequency and it declines small with a rise in frequency and then rises again up to a precise frequency after this frequency, the impedance decreases steeply signifying the presence of two time constants while at 313 and 323 K the change in the impedance values before the sharp decrease is not clear due to the change in the shape of Nyquist plots. Moreover, the impedance of all samples decreases with the increase in temperature at low frequency region.

**Table 4:** Electrochemical parameters and inhibition efficiencies of Al in 0.1 M NaOH solution based on EIS measurements in the absence and presence of 300 ppm of Cefx and Cefz drugs at different temperatures.

	T (K)	Rs (Ω)	C (μF)	CPE		R_ct1_ (Ω)	CPE		R_ct2_ (Ω)	R_T_ (Ω)	η%
				Q-Y_o_ (μF)	n		Q-Y_o_ (mF)	n			
Blank	293	49.35	5.13	–		30.65	5.437	1	13.8	44.45	–
	303	42.02	3.6	–		29.8	6.154	0.268	10	39.8	
	313	0.01	–	972.6	0.026	0.0144	0.00025	1	59.95	59.96	
	323	0.0075	–	4.258	0.745	49.8	598.94	1	3.83	53.63	
300 ppm Cefx	293	61.98	1.95	–		97.12	4.85	1	49.03	146.15	69.58
	303	51.04	2.05	–		58.84	3.306	1	40.57	99.41	59.96
	313	27.25	–	40.55	0.626	64.87	3.696	1	25.045	89.91	33.3
	323	10.57	–	34.51	0.6	63.43	3.44	1	15	78.43	31.62
300 ppm Cefz	293	56.23	2.357	–		73.17	2.955	1	55.25	128.42	65.4
	303	45.79	2.219	–		60.65	4.39	0.984	33.88	94.53	57.9
	313	0.0283	–	56.77	0.526	72.98	4.5735	1	15	87.98	31.85
	323	0.0099	–	17.88	0.576	57.65	4.393	1	9.41	67.06	20.03

Furthermore, the inhibition efficiency of Cefx and Cefz are compared to those of other organic compounds described in the literature as inhibitors for aluminum and its alloys and summarized in Table 5^50–55^. According to Table 5, the proposed drugs have close efficiency or relativity higher than some expired, extracted, synthetic drugs recently reported.

**Table 5:** The inhibition efficiency (η%) of Cefx and Cefz drugs on Al surface in 0.1 M NaOH solution compared to other organic inhibitors in NaOH solution.

Inhibitor	Material	Medium	Inhibitor concentration	Inhibition efficiency (η%)			Ref.
				WL	PDP	EIS	
6-dehydroabietic acylamino sodium (6-DAS	AA2024-T3	0.01 M NaOH	10^–5^–10^–3^ M	96.5	90.01	92.14	^50^
Azo-Schiff base	Aluminum	0.1 M NaOH	1–10 mM	45.5	46.4	49.3	^51^
2,6-dimethylpyridine	Aluminum	1 M NaOH	0.2–0.4 M	–	59.93	55.8	^52^
Ceftriaxone	Aluminum	0.1 M NaOH	50–300 ppm	–	78.4	69.6	This work
Ceftazidime	Aluminum	0.1 M NaOH	50–300 ppm	–	59.5	65.4	This work
sodium silicate and Triton X-100	2024-T3 aluminum alloy	0.01 M NaOH	0.005 M	82.55	86.23	88.73	^53^
Morinda tinctoria leaf extract	Aluminum	0.1 M NaOH	0.2–2.5% v/v	68.9	29	64.7	^54^
Derris indica leaves extract	Aluminum	1 M NaOH	300–1200 ppm	59.8	59.3	60.2	^55^

The inhibition efficiency (η%) listed in the table corresponds to the maximum values obtained in these articles.

### Surface analysis

**Figure 9:**
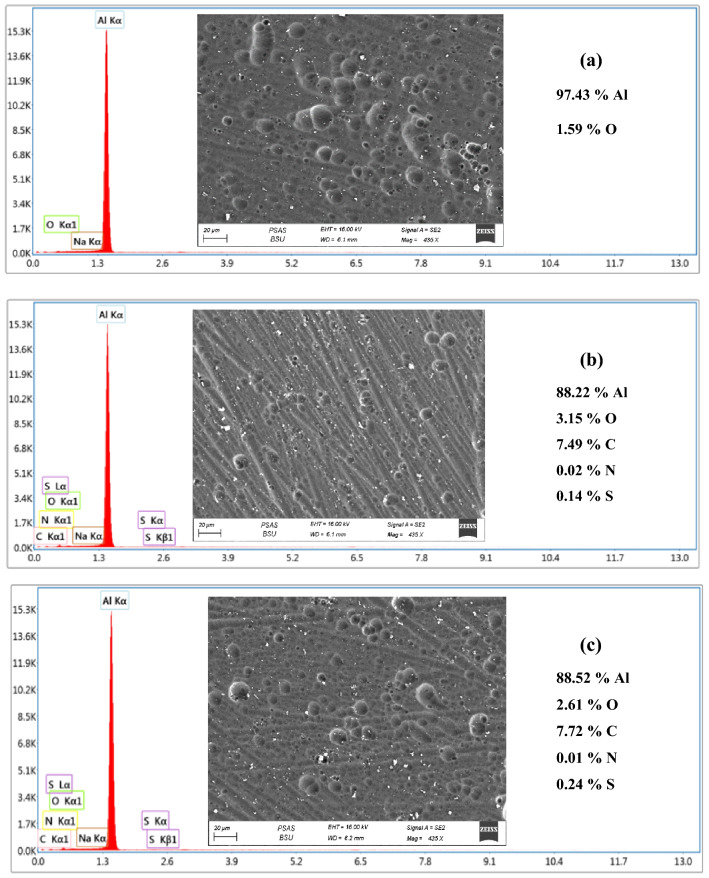
FESEM and EDAX elemental mapping of Al immersed in 0.1 M NaOH solution in the absence and presence of 300 ppm of Cefx and Cefz (**a**) Blank, (**b**) Cefx and (**c**) Cefz at 293 K for 2 h.

The morphology of Al surface in 0.1 M NaOH solutions immersed for 2 h at 293 K in the pure electrolyte and presence of 300 ppm Cefx and Cefz is investigated using FESEM. The FESEM images demonstrate numerous characteristic pores of Al_2_O_3_ in the pure alkaline media with a significant level of roughness caused by the damage of Al surface by the attack of aggressive hydroxide ions while the number and size of these pores decrease in the presence of Cefx and Cefz as shown in Fig. 9. Furthermore, the homogeneity of Al surface in the existence of Cefx is greater than in the presence of Cefz, indicating that Cefx is a more effective inhibitor than Cefz. The improvement of the homogeneity of the Al surface is attributed to the adsorption of these medicines on the Al surface, resulting in the formation of a protective film that shields the metal from the aggressive NaOH solution^5^.

Furthermore, elemental mappings are taken to confirm the distribution and existence of different elements on Al surface after immersion in pure 0.1 M NaOH for 2 h at 293 K in and presence of 300 ppm Cefx and Cefz. Figure 9 reveals the presence of oxygen in the absence of the studied drugs, indicates the presence of Al oxide/hydroxide due to the aggressive attack by OH^−^ ions. While the existence of C, N and S elements following immersion in solutions containing Cefx and Cefz confirmed drug adsorption on the Al surface, the production of the protective adsorbed films that suppressed the aggressive attack between Al surface and OH^−^ ions.

**Figure 10:**
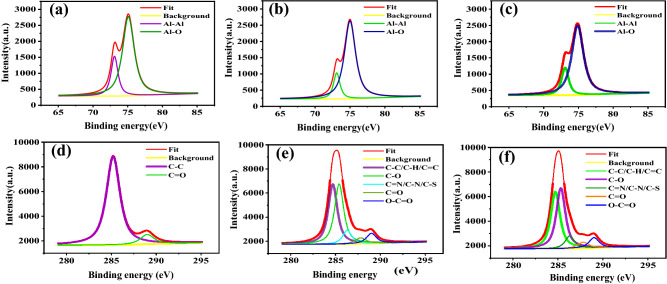
High-resolution XPS spectra carried out in the Al 2p and C 1s binding energy range for Al in 0.1 M NaOH solution in the absence and presence of 300 ppm of Cefx and Cefz at 293 K; Al 2p (**a**) blank, (**b**) Cefx, (**c**) Cefz respectively and C 1s (**d**) blank, (**e**) Cefx, (**f**) Cefz respectively.

XPS is accomplished to investigate the adsorption of Cefx and Cefz on Al surface and examine the chemical composition of the adsorbed film. Table 6 explores the survey spectra inspects the surface composition of Al substrates subjected to the pure NaOH solution and presence of 300 ppm of the examined Cefx and Cefz. The pristine sample has a 24.36 at.% Al and 39.75 at.% O related to the presence of Al oxide/hydroxide. The adsorption of Cefx and Cefz is confirmed by decreasing Al and O contents with increasing the C content, as well as the appearance of new peaks for N and S. High-resolution XPS spectra in the binding energy (BE) range of the Al 2p, C 1s, N 1s, S 2p, and O 1s peaks are presented in Figs. 10, S2, S3, and S4. The deconvolution of the Al 2p (Fig. 10a–c) included two peaks at 73.1 eV and 75 eV for blank, Cefx and Cefz, which are assigned to the Al metal and Al-O respectively^50,56^. The intensity of the Al 2p peak falls somewhat in the presence of the examined drugs as they form a layer on the Al substrate, as shown by the presence of N 1s, S 2p, and an increase in the carbon content on the Al surface. The resolved C 1s spectra of Al for blank solution is deconvoluted into two peaks as shown in Fig. 10d. The first peak at 285.2 eV signified to C–C/C–O/C–H, while the second peak at 289 eV attributed to O–C=O group^57^. The addition of the studied drugs leads to the deconvolution of C 1s peak into five peaks, as shown in Fig. 10e,f. The peak at 284.6 eV was attributed to C–C/C=C/C–H bonds that may have originated from the examined medications, but also to adventitious carbon adsorbed on the Al surface as a result of air exposure^58^. The peaks at 285.5 eV and 286.3 eV are attributed to C–O and C–N/C–S/C=N^59,60^. The two remaining peaks, at 287.5 eV and 289.1 eV, directly correlate to carbonyl and carboxyl functional groups respectively^58^. The XPS spectrum related to the O1s signal is shown as supplementary Fig. S2. The oxygen O1s peak is deconvoluted into two peak singlets. The predominant component at 532.7 eV is ascribed to surface hydroxyl groups in blank samples and oxygen in carbonyl or ester groups inside the adsorbed layer in inhibited solutions while the peak at 531.5 eV is assigned to C–O/Al–O–C^60^. The XPS spectrum related to N 1s (Fig. S3) shows five characteristic peaks at 398.4, 399.5, 400.4, 401.2 eV and 402 eV, corresponding to the C–N, Al–N, C=N, N–C=O and N–N/N–O bonds respectively^61,62^. The S 2p peak of Cefx is deconvoluted into four peaks at binding energies of 162.1 eV, 163.3 eV, 164. eV, and 164.2 eV, assigned to endocyclic (2p3/2, 2p1/2) for thiazine ring, exocyclic C–S–C bond, and C–S–C of thiazole ring, respectively. On the other hand, The figure (Fig. S4) presents the S 2p peak of Cefz is deconvoluted into three peaks due to the absence of an exocyclic S atom^63–66^. The appearance of spectra of N 1s and S 2p for inhibited Al samples reveals that Cefx and Cefz can adsorb on Al surface and increase its corrosion resistance. The atomic percent of elements (Table 6) indicate that the adsorption of the examined medicines on the Al surface occurs in the following order: Cefx > Cefz.

**Table 6:** XPS results of elemental composition (atomic percent) of Al in 0.1 M NaOH in the absence and presence of 300 ppm of Cefx and Cefz at 293 K.

	Surface composition (at%)				
	Al	O	C	N	S
Blank	24.36	39.75	34.76	–	–
Cefx	19.51	38.37	39.09	2.28	0.25
Cefz	19.64	39.18	37.1	1.93	0.35

### Wettability measurements

**Figure 11:**
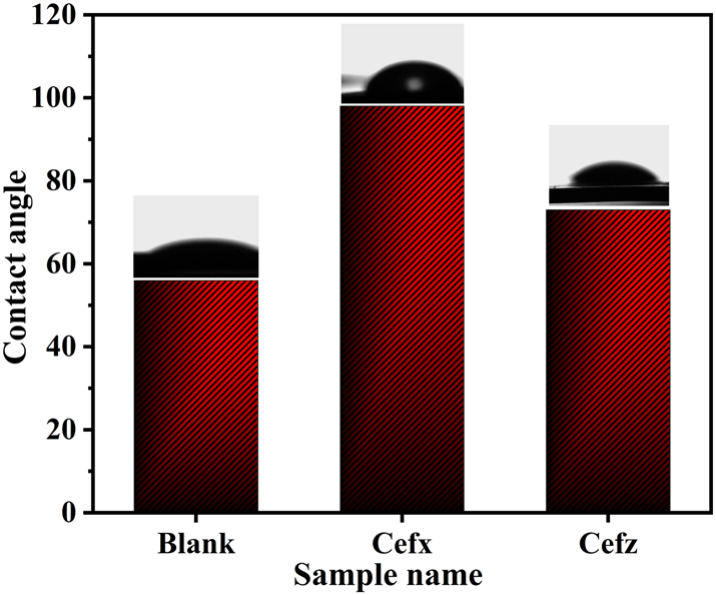
Contact angle measurements of 0.1 M NaOH solutions on Al substrates in the absence and presence of 300 ppm of Cefx and Cefz.

Contact angle measurements proven to be an effective and efficient approach for characterizing the surface wettability behaviour of Al in the absence and existence of 300 ppm of Cefx and Cefz. These findings imply that the hydrophilic character of the blank Al surface with the value of *θ* = 56^o^, undergoes a significant transition to hydrophobic nature, with values of 98° and 73^o^ for the Cefx and Cefz, respectively, as shown in Fig. 11. The hydrophobicity nature of Al surface in the presence of the studied drugs can be correlated to the adsorption of the hydrophobic groups in their molecular structures. Therefore, it decreases the adhesion contact with Al surface and enhances its corrosion resistance^67–71^. This increase in hydrophobicity is consistent with the predicted inhibitory efficiencies, which are listed in the following order: Cefx > Cefz.

### Adsorption isotherms

**Figure 12:**
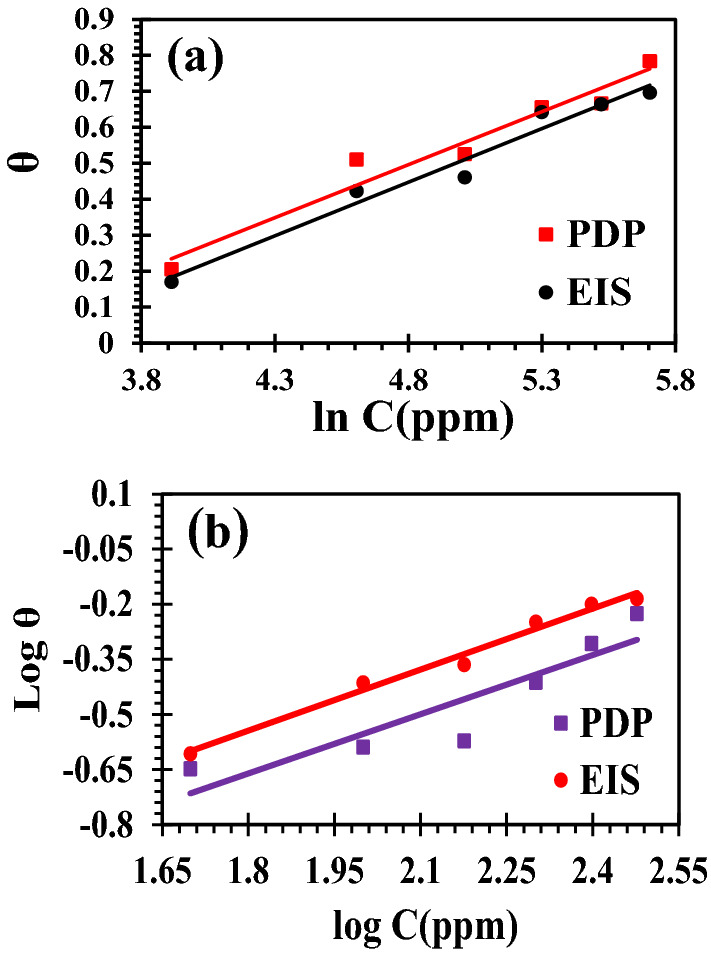
Adsorption isotherms based on PDP and EIS measurements for Al in 0.1 M NaOH containing different concentrations of Cefx and Cefz at 293 K; (**a**) Temkin isotherm for Cefx and (**b**) Freundlich isotherm for Cefz.

Adsorption isotherms give significant information regarding the interaction between the Al/electrolyte interface and the researched medicines, depending on the charges dispersed throughout their chemical structures^72^. As depicted in Fig. 12, various isotherms are investigated using experimental data from EIS, and PDP measurements at 293 K. Temkin adsorption isotherm is found to be the best fit one for Cefx while Freundlich adsorption isotherm fits Cefz well as^73,74^. Table 7 shows the computed values of K_ads_ and ΔG^o^_ads_ for Cefx and Cefz based on the best fitting adsorption isotherms. The results indicate that the adsorption of Cefx and Cefz on the Al surface is spontaneous and mixed, with physisorption dominating both drugs. This is due to the electrostatic interaction between the charged Al surface and charged drug molecules which is characterized by the values of ΔG^o^_ads_^72,75^. The greater K_ads_ and more negative ΔG^o^_ads_ values for Cefx explain the drug's intense and impulsive adsorption on the Al surface compared to Cefz, resulting in a better protection efficiency that fits with earlier findings. The higher adsorption of Cefx can be explained to the presence of dioxo-methyl-triazin-sulfanyl moiety in its structure which is confirmed by theoretical calculations.

**Table 7:** Thermodynamic parameters of adsorption process based on the best fitted adsorption isotherm calculated from PDP and EIS measurements at 293 K.

Inhibitor	PDP		EIS	
	K_ads_ (ppm^−1^)	ΔG^o^_ads_ (kJ/mol)	K_ads_ (ppm^−1^)	ΔG^o^_ads_ (kJ/mol)
Cefx	0.0443	− 26.062	0.0369	− 25.617
Cefz	0.0235	− 24.517	0.0286	− 24.99-

### Thermodynamic activation parameters

**Figure 13:**
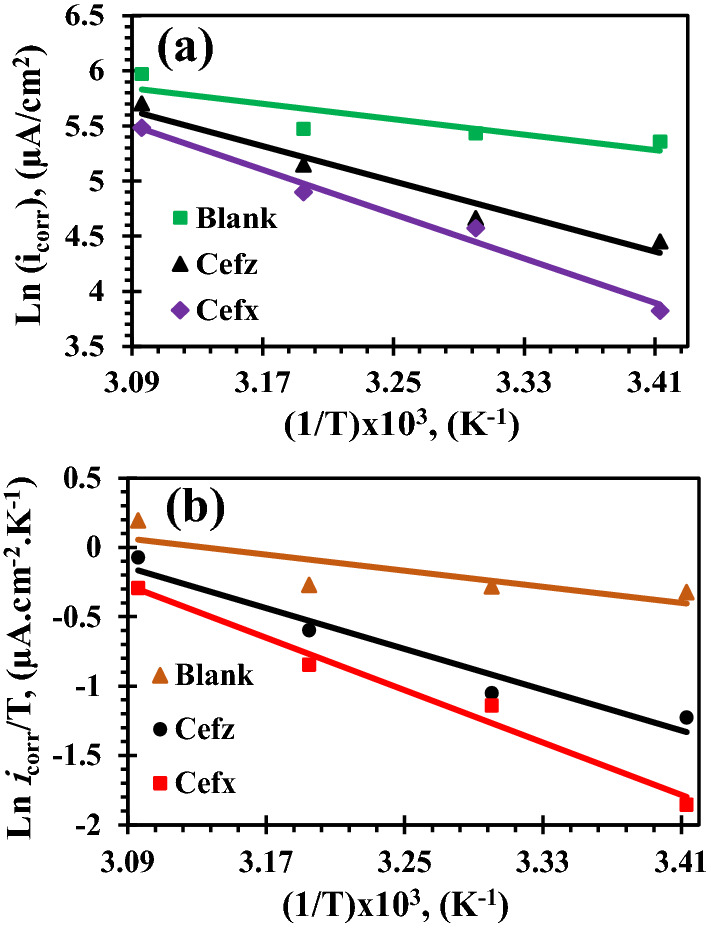
Kinetic thermodynamic parameters of Al in 0.1 M NaOH based on PDP measurements in the absence and presence of 300 ppm of Cefx and Cefz; (**a**) Arrhenius plots and (**b**) Transition state equation.

Thermodynamic activation parameters are determined and summarised in Table 2 for the pure electrolyte, and the existence of 300 ppm of Cefx and Cefz are calculated and tabulated in Table 2 using Arrhenius and transition state equations as follows^76^:

16 $${\text{Ln }}\left( {{\text{CR}}\sim {\text{ I}}_{{{\text{corr}}}} } \right) = {\text{ Ln A}} - {\text{ E}}_{{\text{a}}} /{\text{RT}}$$

17 $${\text{Ln }}\left( {{\text{CR}}\sim {\text{ I}}_{{{\text{corr}}}} /{\text{T}}} \right) = {\text{ Ln}}\left( {{\text{R}}/{\text{Nh}}} \right) + \Delta {\text{S}}^{*}/{\text{R }} -\Delta {\text{H}}^{*}/{\text{RT}}$$

where A is the Arrhenius constant, R represents the universal gas constant, T donates the absolute temperature, n is Avogadro's number and h refers to plank's constant. ΔH* and ΔS* are the dissolution process's activation enthalpy and entropy. The corrosion rate (CR) is directly proportional to the corrosion current density (I_corr_), and E_a_ denotes the activation energy. Figure 13a represents the linear relationships of Ln (I_corr_) with 1/T for Al in pure 0.1 M NaOH and presence of 300 ppm of Cefx and Cefz. The values of E_a_ are calculated and listed in Table 2. Data show that the values of E_a_ in the presence of studied drugs are higher than that in blank solution which indicate that rate of Al dissolution decreases in presence of Cefx and Cefz. Moreover, the values of E_a_ increase in the following order Cefz < Cefx which indicate that the rate of Al dissolution in the presence of Cefx is lower than that in the presence of Cefz and hence Cefx is more efficient inhibitor than Cefz. Figure 13b represents the relation between Ln (I_corr_/T) and 1/T, from which the values of ΔH* and ΔS* are calculated and summarized in Table 2. The obtained results display that ΔH* values are positive indicating the endothermic nature of Al dissolution process. Higher values of ΔH* in the presence of Cefx and Cefz compared to inhibited free solution enhance the corrosion resistance of Al in 0.1 M NaOH^76^, as a result of their adsorption on its surface forming an energy barrier which is higher for Cefx in comparison with Cefz. Furthermore, negative values of ΔS* suggest that association rather than dissociation is the rate-determining step to produce activated complex, resulting in a reduction in disturbance^76^. Furthermore, the higher ΔS* values in the presence of Cefx and Cefz may be ascribed to an increase in randomness at the Al/solution interface after their adsorption, which can be explained by the increased number of water molecules desorbed off the Al surface^77^. Moreover, the more positive value of ΔS* for Cefx (− 78.49 kJ/mole) compared to Cefz (− 104.19 kJ/mole) implying that the higher protection efficiency is for Cefx.

### Quantum chemical calculations

**Figure 14:**
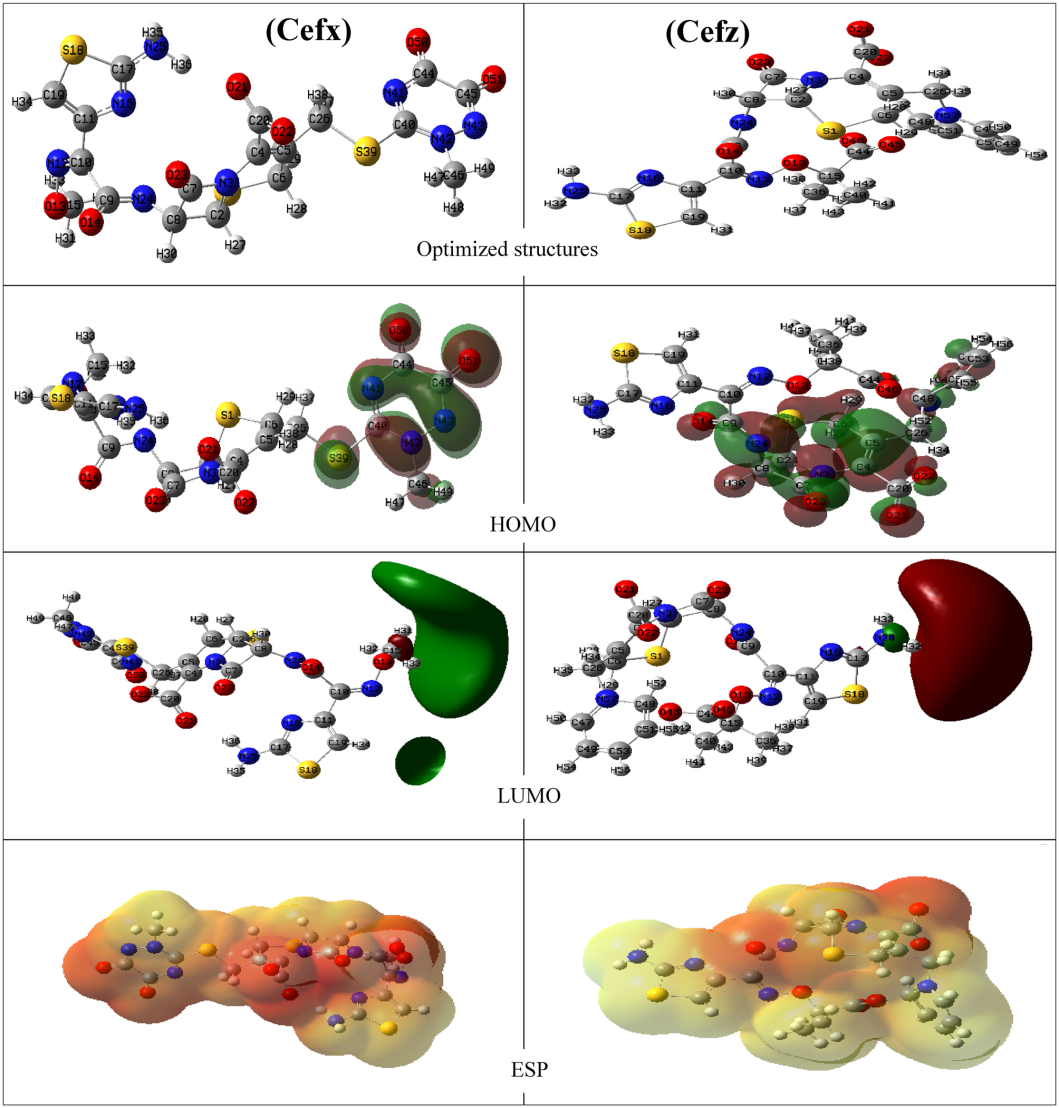
The optimized structures, HOMOs, LUMOs and electrostatic potential (ESP) structures of Cefx and Cefz drugs using HF /6–311++G (d, p) method.

The corrosion inhibition efficiency of the Cefx and Cefz are also investigated by doing QC based on HF. The adjusted molecular structures, HOMO (the highest occupied molecular orbital), LUMO (the lowest unoccupied molecular orbital) and molecular electrostatic potentials (ESP) of both drugs are depicted in Fig. 14. The quantum chemical factors, including *E*_HOMO_, *E*_LUMO_, HOMO–LUMO energy gap (ΔE), ionization energy (I), electron affinity (A), electronegativity (χ), chemical hardness (η), softness (σ) and fraction of electron transferred (ΔN) are used in corrosion studies to evaluate the inhibition performances of the studied drugs. E_HOMO_ is correlated to the electron donating ability of the molecules. The higher the E_HOMO_, the higher the electron transfer from inhibitor to the unoccupied molecular orbital of the metal that facilitates the adsorption and therefore enhances inhibition efficiency^78^. ΔE is an important parameter considered in corrosion science as we know the reactivity of inhibitor molecules through it. As ΔE decreases, the inhibition efficiency of inhibitors increases. Ionization energy (I) is another important parameter of chemical reactivity of atoms and molecules. Small (I) implies high reactivity of molecules while high (I) indicates the inertness of the molecules and hence low inhibition efficiency^79^. The electron affinity (A) denotes the intensity of inhibitor adsorption on the surface of metal sample; a high negative value of (A) indicates that the inhibitor molecule firmly absorbs on the metal surface, forming a protective coating on the surface^80^. The electronegativity is very important descriptor as it represents electron pulling power of the molecules. High χ values suggest that the molecules cannot give electrons easily^81^. Furthermore, low value of ΔE indicates that the molecule is soft and polarizable. Polarizable and soft molecules give electrons to metal easier than the unpolarizable and hard ones^82^. Hardness and softness are crucial factors because they measure a molecule's reactivity and stability^82^. The inhibitory efficiency is proportional to the percentage of electrons transferred (ΔN) and the positive ΔN values imply that the electrons are moved from the inhibitor to the metal, while negative ΔN values indicate that electrons are transferred from the metal to the inhibitor molecule^83^. The inhibitory efficacy of inhibitors rises as ΔN values increase owing to an increase in electron donating between the examined medicines and the Al surface.

It is clear from Fig. 14 that HOMO of the studied drugs is focused on oxygen, nitrogen, and Sulphur of cephalosporin group for Cefz and dioxo-methyl-triazine sulfanyl group for Cefx. The higher inhibition efficiency of Cefx compared to Cefz is correlated to its higher values of E_HOMO,_ σ and ΔN with lower values of ΔE, I, A, χ and η as shown from Table 8. The above results can be illustrated to the presence of dioxo-methyl-triazine sulfanyl group in Cefx molecular structure.

**Table 8:** Calculated quantum chemical parameters for Cefx and Cefz based on HF/6–311++G (d, p) method.

Property	Cefx	Cefz
E_HOMO_ (eV)	− 0.787168	− 2.702592
E_LUMO_ (eV)	5.938032	4.106928
ΔE (eV)	6.7252	6.80952
I (eV)	0.787168	2.702592
A (eV)	5.938032-	− 4.106928
χ (eV)	− 2.575432	− 0.702168
η (eV)	3.3626	3.40476
σ (eV^−1^)	0.29738	0.293706
ΔN	0.863235	0.57745

Molecular electrostatic potential (ESP) maps are very important parameters as they provide a visual method to know the parts of molecules on which the electron density is higher than other parts to determine the reactive parts in the molecules^82^. Different colors reflect different ESP values. The blue indicates the area with the highest positive ESP, the red indicates the region with the most negative ESP, and the green indicates the region with zero ESP. It can be shown from Fig. 14 that Cefz has red regions less than that of Cefx. In additions the figure shows that the electron rich centers are referred to the oxygen of dioxo-methyl-triazine sulfanyl group and other functional groups in Cefx while oxygen of carboxylate and amide groups in Cefz. It can be concluded that the presence of dioxo-methyl-triazine sulfanyl group in Cefx drug increases its electron density, so increases the electrostatic attraction with Al surface and consequently its inhibition efficiency. These findings agree well with experimental data.

### Monte Carlo simulations

**Figure 15:**
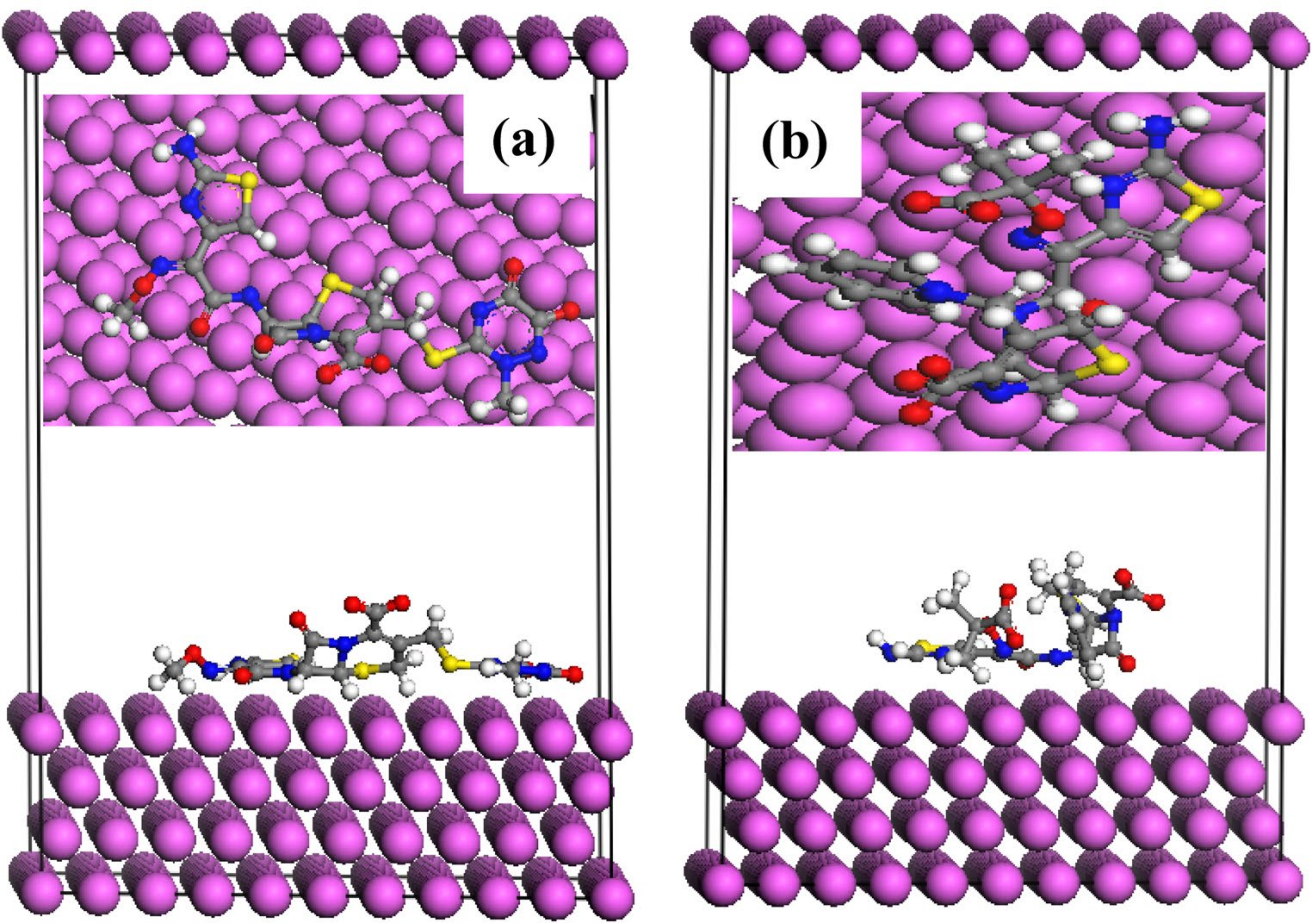
The lowest-energy geometry of the adsorbed (**a**) Cefx and (**b**) Cefz on the Al (111) surface as obtained from MC simulation.

MC simulations explore the interaction between the studied inhibitors and Al surface. The side and top views of the stable adsorption configuration of a single molecule of Cefx and Cefz with Al (111) surface are shown in Fig. 15. It is observed from the figure that the surface adsorption of Cefx molecule preferably happened by flat type molecular orientation and parallel to Al surface. This orientation allows its active centers to react effectively with the Al surface leading to higher protection efficiency than Cefz molecule. On the hand, the adsorption energies of Cefx and Cefz are − 239.81 and − 156.55 kcal/mol, respectively. Cefx's largest negative adsorption energy value reflects its strongest spontaneous adsorption on Al when compared to Cefz, and hence its better inhibitory effectiveness^81,83^.

### Molecular dynamics simulations

**Figure 16:**
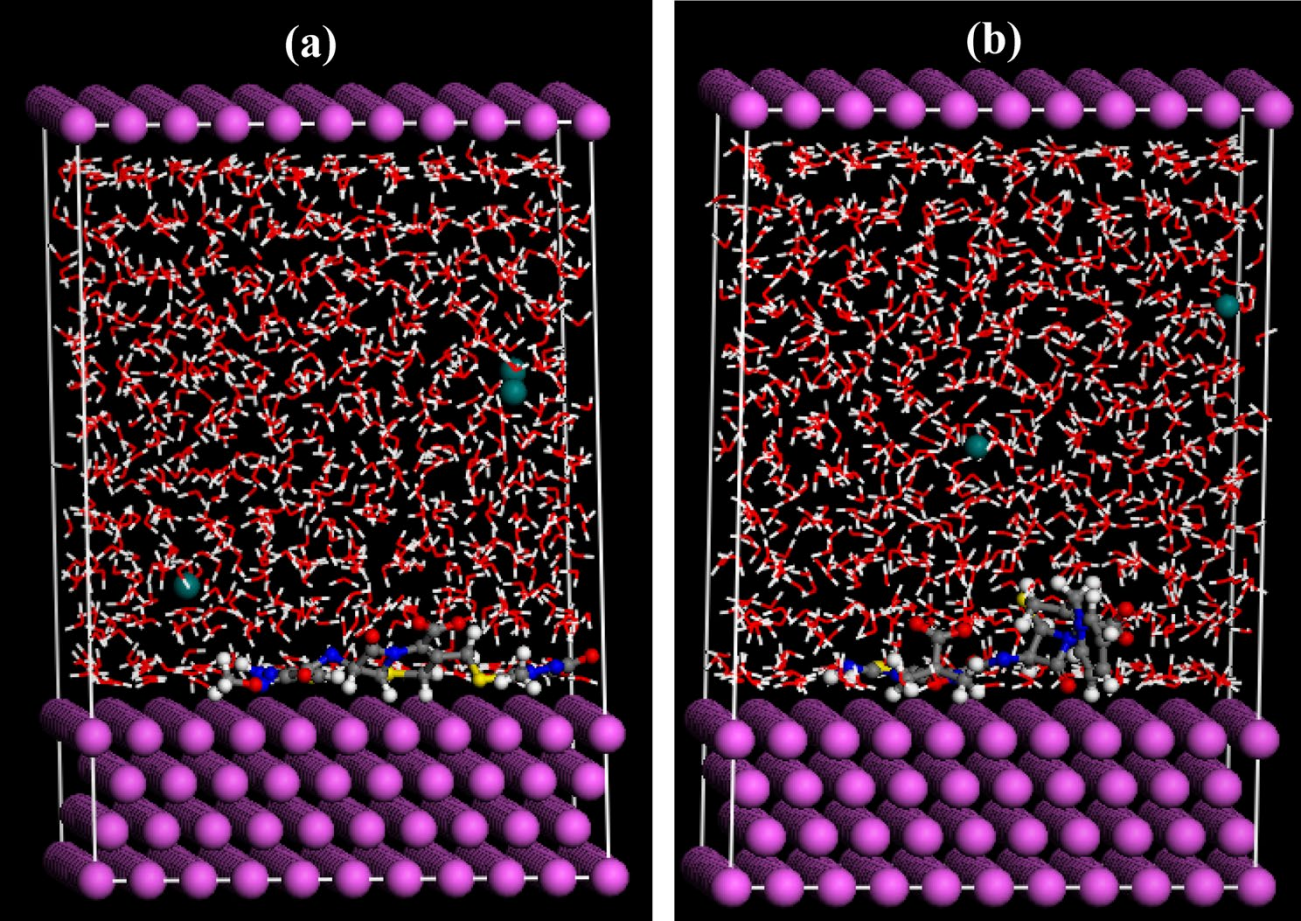
MD Snapshots of the most stable low energy configuration for the adsorption of Cefx and Cefz drugs on Al (111)/950H_2_O interface (**a**) Cefx and (**b**) Cefz.

MD simulations are performed to explore the interactions of the investigated drugs with Al surface in presence of water molecules. The side views of the final MD snapshots of the Cefx and Cefz adsorbed on the surface of Al (111) with the presence of 950 H_2_O molecules are shown in Fig. 16. This image clearly shows that the examined drugs are situated extremely close to the metal surface, indicating that the affinity of Cefx and Cefz for the Al surface is strong even in the presence of solvent molecules. This validates the adsorption of these medicines on the Al surface, protecting it against corrosion.

Radial distribution function (RDF) is applied to calculate the distances between the atoms of the studied drugs and the Al surface to highlight the type of the adsorption process. If the value of the interatomic distance (r) is within the range of 1–3.5 Å, then the chemical bonds are favorably present, while physical interactions have been observed at r > 3.5 Å^84^. Figure S5 shows RDF for oxygen atoms of the active functional groups for both Cefx and Cefz drugs and top layer of Al (111) surface. It is observed from the figure that, the distance found for O_13_ (2.71 Å), O_14_ (2.93 Å), O_21_&O_22_ (6.07 Å), O_23_ (5.35 Å) and O_50_ &O_51_ (3.33 Å) with respect to Al(111) surface in case of Cefx, while the distances for Cefz are O_13_ (2.83 Å), O_14_ (3.25 Å), O_21_&O_22_ (4.47 Å), O_23_(2.75 Å) and O_45_&O_46_ (5.67 Å). These results suggesting a mixed type adsorption of these drugs on Al surface, that agree with the values of ΔG^o^_ads_ calculated from the best fit experimental adsorption isotherms.

## Conclusion

In this work, various doses of anionic states of Cefx and Cefz medicines are tested as corrosion inhibitors for Al in 0.1 M NaOH solution at 293 K. The electrochemical results show that Cefx and Cefz are effective Cathodic inhibitors. Furthermore, the inhibitory efficiencies rise with increasing concentrations, with the greatest percent (78.4 percent) recorded at 300 ppm of Cefx compared to 59.5 percent at 300 ppm of Cefz. The activation energy barrier of Al dissolving in 0.1 M NaOH is low compared to that in the presence of Cefx, which is greater than that of Cefz, suggesting a reduction in corrosion rate in the presence of the examined medications. Surface analysis and wettability studies show the existence of adsorbed hydrophobic layers of Cefx and Cefz on the Al surface. Cefz obeys the Freundlich isotherm with spontaneous and mixed type adsorption, while Cefx obeys the Temkin isotherm with spontaneous and mixed type adsorption. MC/MD simulations demonstrate that Cefx has an adsorbed flat type molecular orientation parallel to the Al surface, indicating that it has a greater protective effect than Cefz medication.

## Supplementary Information

Supplementary Figure S1.

Supplementary Figure S2.

Supplementary Figure S3.

Supplementary Figure S4.

Supplementary Figure S5.
