# Supplementary material for: Electrochemical and computational estimations of cephalosporin drugs as eco-friendly and efficient corrosion inhibitors for aluminum in alkaline solution
Source: Sci Rep. 2022 Aug 3;12:13333. doi: 10.1038/s41598-022-17423-5 (PMC9349255; doi:10.1038/s41598-022-17423-5)
Supplement: Supplementary file 1 — Supplementary Figure S1. [file 41598_2022_17423_MOESM1_ESM.docx]

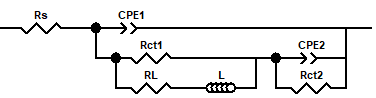


**(a) EC_2_**

**Fig.S1.** Equivalent circuit (EC) and Bode plots of Al in 0.1M NaOH solution at E_OCP_ in the absence and presence of 300 ppm of Cefx and Cefz drugs at different temperatures (a) EC_2_ at 313 and 323 K, (b), (c) and (d) Bode plots of blank, Cefx and Cefz respectively.
