# Supplementary material for: Electrochemical and computational estimations of cephalosporin drugs as eco-friendly and efficient corrosion inhibitors for aluminum in alkaline solution
Source: Sci Rep. 2022 Aug 3;12:13333. doi: 10.1038/s41598-022-17423-5 (PMC9349255; doi:10.1038/s41598-022-17423-5)
Supplement: Supplementary file 2 — Supplementary Figure S2. [file 41598_2022_17423_MOESM2_ESM.docx]

**Figure S2**: High-resolution XPS spectra carried out in O1s binding energy range for Al in 0.1M NaOH Solution at 293 K (a) Blank, (b) 300 ppm Cefx, (c) 300 ppm Cefz.
