# Supplementary material for: Electrochemical and computational estimations of cephalosporin drugs as eco-friendly and efficient corrosion inhibitors for aluminum in alkaline solution
Source: Sci Rep. 2022 Aug 3;12:13333. doi: 10.1038/s41598-022-17423-5 (PMC9349255; doi:10.1038/s41598-022-17423-5)
Supplement: Supplementary file 3 — Supplementary Figure S3. [file 41598_2022_17423_MOESM3_ESM.docx]

**Figure S3:** High-resolution XPS spectra carried out in N 1s binding energy range for Al in 0.1M NaOH Solution in the presence of 300 ppm of the studied drugs at 293 K; (a)Cefx and (b)Cefz.
