# Supplementary material for: Electrochemical and computational estimations of cephalosporin drugs as eco-friendly and efficient corrosion inhibitors for aluminum in alkaline solution
Source: Sci Rep. 2022 Aug 3;12:13333. doi: 10.1038/s41598-022-17423-5 (PMC9349255; doi:10.1038/s41598-022-17423-5)
Supplement: Supplementary file 4 — Supplementary Figure S4. [file 41598_2022_17423_MOESM4_ESM.docx]

**Figure S4**. High-resolution XPS spectra carried out in S 2P binding energy range for Al in 0.1M NaOH Solution in the presence of 300 ppm of the studied drugs at 293 K; (a) Cefx and (b) Cefz.
