# Supplementary material for: Electrochemical and computational estimations of cephalosporin drugs as eco-friendly and efficient corrosion inhibitors for aluminum in alkaline solution
Source: Sci Rep. 2022 Aug 3;12:13333. doi: 10.1038/s41598-022-17423-5 (PMC9349255; doi:10.1038/s41598-022-17423-5)
Supplement: Supplementary file 5 — Supplementary Figure S5. [file 41598_2022_17423_MOESM5_ESM.docx]

**Fig. S5.** RDFs for the adsorption of Cefx and Cefz on Al(111) surface in water obtained from MD simulation: (a) oxygen atoms of Cefx, (b)oxygen atoms of Cefz.
